# Tuberculose multifocale révélée par une pancytopénie: à propos d’un cas

**DOI:** 10.11604/pamj.2018.31.92.17046

**Published:** 2018-10-05

**Authors:** Nabil Tiresse, Mohamed Allaoui

**Affiliations:** 1 Service de Pneumologie, Hôpital Militaire d’Instruction Mohamed V, Rabat, Maroc; 2 Service d’Anatomie Pathologique, Hôpital Militaire d’Instruction Mohamed V, Rabat, Maroc

**Keywords:** Biopsie ostéo-médullaire, Gene Xpert, miliaire, cytolyse, Osteomedullary biopsy, Genexpert TB test, miliary, cytolysis

## Abstract

We here report the case of a 58-year old patient admitted with respiratory distress, oxygen desaturation 65% while breathing ambient air and fever 38.5°C. Blood count showed pancytopenia. Ionogram objectified slight hepatic cytolysis and chest X-ray showed bilateral miliaria. BK research using sputum was negative as well as Genexpert TB test. Anatomopathological examination of the osteomedullar biopsy revealed epitheliogigantocellular granuloma without caseous necrosis and polymerase chain reaction (PCR) showed mycobacterium tuberculosis in osteomedullary biopsy. Liver biopsy showed epithelioid granuloma without caseous necrosis. The diagnosis of multifocal tuberculosis with pulmonary, hematopoietic and hepatic impairment was retained. Miliary Tuberculosis is a severe form of tuberculosis. Bacilloscopies are often negative and the diagnosis can be retained based on other sampling, including cerebrospinal fluid sampling and osteomedullary biopsy. Different kinds of biopsies enable diagnosis in rare cases. PCR, a technique used in molecular biology, has enabled to shorten the window period from disease onset to diagnosis as well as treatment implementation. Miliary tuberculosis can be life-threatening; therefore, sometimes, treatment must be administered before certain diagnosis.

## Image en médecine

Patiente de 58 ans, admise dans un tableau de polypnée avec signes de lutte respiratoire et désaturant à 65% à l’air ambiant et une fièvre à 38,5°C, l’hémogramme trouve une pancytopénie, l’ionogramme objctive une légère cytolyse hépatique et la radio des poumons objective un aspect de miliaire bilatérale. Les recherches de BK dans les expectorations étaient négatives ainsi que le Gene Xpert. L’étude anatomo-pathologique de la biopsie ostéomédullaire montre un granulome épithélio-giganto-cellulaire sans nécrose caséeuse et la PCR met en évidence le mycobacterium tuberculosis dans le prélèvement de biopsie ostéo-médullaire. Une biopsie hépatique a mis en évidence un granulome épithélioïde sans nécrose caséeuse. Le diagnostic de tuberculose multifocale avec atteinte pulmonaire, hématopoïétique et hépatique était retenu. La miliaire tuberculeuse est l’une des atteintes sévères dans le cadre de la tuberculose, les bacilloscopies sont souvent négatives et le diagnostic peut être retenu par d’autres prélèvements notamment l’étude du liquide céphalo-rachidien et la biopsie ostéomédullaire. La biopsie d’autres organes peut apporter le diagnostic dans de rares cas. L’avènement de la PCR comme technique de biologie moléculaire a permis de raccourcir le délai de diagnostic et donc la mise en route du traitement qui parfois doit être administré avant même d’obtenir la certitude diagnostique compte tenu de la mise en jeu du pronostic vital au cours de ces atteintes sévères.

**Figure 1:**
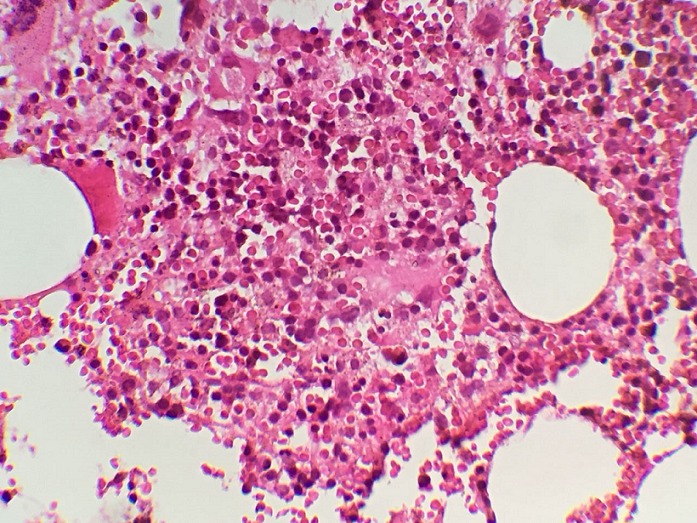
moelle osseuse siège de granulome épithélio-giganto-cellulaire

